# Consumer Awareness of Food Defense Measures at Food Delivery Service Providers and Food Manufacturers: Web-Based Consumer Survey Study

**DOI:** 10.2196/44150

**Published:** 2023-08-24

**Authors:** Manabu Akahane, Yoshiyuki Kanagawa, Yoshihisa Takahata, Yasuhiro Nakanishi, Takemi Akahane, Tomoaki Imamura

**Affiliations:** 1 Department of Health and Welfare Services National Institute of Public Health Wako Japan; 2 Department of Public Health, Health Management and Policy Nara Medical University Kashihara Japan; 3 Department of Management, Food Business Course Osaka Seikei University Osaka Japan; 4 Department of Gastroenterology Nara Medical University Kashihara Japan

**Keywords:** food defense, health hazards, intentional contamination, foreign substances, food delivery service

## Abstract

**Background:**

Various stages of the food chain, from production to processing to distribution, can impact food safety. The concept of “food defense” has emerged as a countermeasure against intentional contamination of food with foreign substances. Although knowledge of food hygiene is common among consumers, there are currently no reports of consumer surveys on food defense.

**Objective:**

This study aims to investigate consumer awareness of food defense and food safety. We analyzed the results focusing on how consumers behave when they find abnormalities in food to further our knowledge on promoting food defense measures.

**Methods:**

Participants completed a web-based questionnaire that included items related to awareness of food safety and food defense, as well as actions to be taken in cases of food abnormalities, such as contamination by foreign substances, the presence of a bad smell in purchased food, and the inclusion of extra items not selected by the individual. The participants were asked to indicate their preference among the 5 suggested actions in each case using a 6-point Likert scale. Data analysis involved aggregating responses into binary values. Stepwise linear regression analysis was conducted to examine the relationship between selected actions and questionnaire items, such as sex, age, and personality.

**Results:**

A total of 1442 respondents completed the survey, and the majority of participants placed importance on food safety when making food purchases. The recognition of each term was as follows: 95.2% (n=1373) for “food security and safety,” 95.6% (n=1379) for “food hygiene,” and 17.1% (n=247) for “food defense.” The percentages of those who answered that they would “eat without worrying” in the case of “contamination by foreign substances,” “bad smell,” or “including unpurchased product” in the frozen food they purchased were 9.1% (n=131), 4.8% (n=69), and 30.7% (n=443), respectively. The results showed that contacting the manufacturer was the most common action when faced with contaminated food or food with a bad smell. Interestingly, a significant percentage of respondents indicated they would upload the issue on social networking sites. Logistic regression analysis revealed that male participants and the younger generation were more likely to choose the option of eating contaminated food without worrying. Additionally, the tendency to upload the issue on social networking sites was higher among respondents who were sociable and brand-conscious.

**Conclusions:**

The findings of this study indicate that if food intentionally contaminated with a foreign substance is sold and delivered to consumers, it is possible consumers may eat it and experience health problems. Therefore, it is crucial for not only food manufacturers but also food delivery service providers to consider food defense measures such as protecting food from intentional contamination. Additionally, promoting consumer education and awareness regarding food defense can contribute to enhancing food safety throughout the food chain.

## Introduction

In recent years, thorough measures to improve food safety in the food chain for consumers have become a necessity [1,2]. The food chain is the flow of food supply from primary production (production of raw materials) to consumers, which travels from production to processing to distribution to storage and finally to sales. As all factors at each stage of the food chain can affect food safety, necessary actions must be taken [3,4]. To avoid the risk of food-related health hazards, it is necessary for businesses to promote food protection measures and for consumers at the end of the food chain to adopt the appropriate measures, such as food hygiene measures [5].

With frequent incidents of falsified expiration dates and contamination of food with foreign substances, the interest in food safety has increased considerably [6-9]. A typical example of food contamination by foreign substances in Japan was the health scare caused by frozen dumplings from China that occurred in December 2007. A total of 10 people complained of diarrhea and other symptoms of food poisoning, which led to the detection of an insecticide (methamidophos) in the imported dumplings. Then, 6 years later, another incident occurred in which frozen foods produced at a factory in Gunma prefecture in Japan were contaminated with a type of pesticide (malathion) [10], shattering consumers’ vague notion of “domestic products being safe.”

Both the mentioned examples are criminal incidents where employees involved in food production intentionally mixed foreign substances into the food. These incidents show the reality of “using food to cause health problems” and “contaminating food with foreign substances out of dissatisfaction with the company.” Moreover, they clearly demonstrate that food safety measures that only assume external crimes are insufficient. As a result, the term *food defense* has been repeatedly used in the media and is now a concept common not only in the food industry but also among consumers. Food defense is a countermeasure against food contamination caused by the intentional introduction of foreign substances [11,12]. Therefore, food-related companies and businesses must implement not only food hygiene measures but also food defense measures to ensure food safety. Food defense refers to practicing “safety management to protect against attacks on food, such as intentional contamination of food with foreign substances or contaminants” [13-15].

Although the concept of food hygiene is widely known to consumers, no consumer surveys on food defense have been reported. Therefore, in this study, we conducted a web-based questionnaire survey to investigate consumer awareness of food defense and food safety. We analyzed the results with a focus on how consumers behave when they find abnormalities in the food they have purchased to further our knowledge on promoting food defense measures in the future.

## Methods

### Ethics Approval

The Ethical Committee of the National Institute of Public Health approved the study design (NIPH-IBRA#12302). All participants provided informed consent for data collection and storage. Written informed consent for study participation was obtained at the time of registration. In cases where participants were aged 18 years or younger, a consent screen was displayed to obtain permission from their parents or guardians. The web-based questionnaire survey was conducted by an authorized survey company in compliance with personal information protection regulations. After the questionnaire survey was completed, we obtained anonymized data from the company.

### Study Design and Participants

This study was conducted as a cross-sectional survey in January 2021 as a web-based panel survey. Initially, a screening survey was conducted among monitors registered with the panel survey company to recruit participants for this study. The research company randomly sampled registered participants aged between 15 and 79 years and emailed them regarding their interest in participating in our survey. When the number of participants in each group (eg, sex and age) reached the target sample size (103), the subject registration for this study was closed. Subsequently, participants were asked to answer a questionnaire on the web-based survey screen. Upon completion of the survey, participants received a small cash reward.

### Web-Based Questionnaire Survey Items

In this study, a custom-designed questionnaire survey was developed in collaboration with an expert in food defense research. A preliminary pilot survey was conducted involving 15 individuals (representing approximately 1% of the total sample size for the questionnaire) to assess the questionnaire’s completeness, clarity, and consistency. Adjustments were made to the questionnaire based on the pilot test findings to enhance its coherence. The web-based survey screens were created by a dedicated survey company.

Age, sex, household income, and residential area were recorded during the survey panel monitors’ registration. In this survey, participants responded to items related to food safety, food defense, awareness of food protection, and actions to be taken in 3 cases of abnormalities, such as contamination of foreign substances, bad smell of purchased food, and extra items which the customer had not selected. For the 3 cases, respondents were asked whether they would take the suggested actions. The questionnaire items shown in Textbox 1 were analyzed in this study. For each item (with some exceptions), the respondents were asked to answer on a 6-point Likert scale: 1=strongly disagree, 2=disagree, 3=somewhat disagree, 4=somewhat agree, 5=agree, and 6=strongly agree.

**Questionnaire items analyzed in this study.**

**Please tell us about yourself.**

1. I am diligent
2. I am sociable
3. I have a strong sense of responsibility
4. I have a strong sense of morality
5. I am cooperative
6. I am honest

**The following are important for you when buying food.**

1. Price
2. Brand
3. Manufactured domestically
4. Reputation (word of mouth)
5. Customer service/troubleshooting
6. Safety

**Have you heard of the following terms? (Yes/no)**

1. Food security and safety
2. Food hygiene
3. Intentional contamination of food
4. Food terrorism
5. Food defense

**The frozen food you purchased is contaminated with foreign substances (metal, hair, etc.). You...**

1. Eat it without worrying
2. Contact the manufacturer
3. Contact the shop from where you purchased it
4. Upload it on SNS (social networking sites)
5. Dispose of it

**The frozen food you purchased has a bad smell (rotten, chemical, etc.). You...**

1. Eat it without worrying
2. Contact the manufacturer
3. Contact the shop from where you purchased it
4. Upload it on SNS
5. Dispose of it

**You find extra item(s), which you had not selected, in the food parcel delivered to you. You...**

1. Eat it without worrying
2. Contact the shop from where you had ordered it
3. Upload it on SNS
4. Dispose of only the extra item(s)
5. Dispose of all the items

### Data Analysis

We combined the data into 2 binary values: “disagree” for 1=strongly disagree, 2=disagree, and 3=somewhat disagree, and “agree” for 4=somewhat agree, 5=agree, and 6=strongly agree.

To estimate the relationship between the actions that the respondents selected and the items of the questionnaire, a stepwise linear regression analysis was conducted. The dependent variable included the answers “agree” or “disagree,” while the independent variables included categories such as sex, age, household income, residential area, personality, and temperament of participants (eg, diligent, sociable, cooperative, and honest, or having a strong sense of responsibility or morality), and what is important when purchasing food (eg, price, brand, manufactured domestically, reputation or word of mouth, and safety) as determined from the questionnaire.

Statistical analyses were conducted using SPSS (version 27.0; IBM). The level of significance was set at *P*<.05.

## Results

### Participants

A total of 1442 respondents (103 men and 103 women in each age group) who answered all questions were included in the analysis. Of the respondents, 53.5% were married and 51% (n=735) had children. The regions of residence were as follows: Hokkaido, 4.6% (n=66); Tohoku, 5% (n=72); Chubu, 16.5% (n=23); Kanto, 40.2% (n=58); Kinki, 19.1% (n=275); Chugoku, 4.4% (n=63); Shikoku, 2.1% (n=30); and Kyushu, 8% (n=115).

The responses to the questionnaire item “The following are important for you when buying food,” showed that more than 93.6% (n=1350) of the respondents tended to place importance on safety, with 37.7% (n=544) strongly agreeing, 33.6% (n=735) agreeing, and 22.3% (n=322) somewhat agreeing. The recognition of each term was as follows: 95.2% (n=1373) for “food security and safety,” 95.6% (n=1379) for “food hygiene,” 17.6% (n=254) for “intentional contamination of food,” 46.3% (n=668) for “food terrorism,” and 17.1% (n=247) for “food defense.”

### Evaluation Outcomes

The results of the questions “The frozen food you purchased is contaminated with foreign substances (metal, hair, etc.). You...” were as follows: 9.1% (n=131) for “eat it without worrying,” 77.6% (n=1119) for “contact the manufacturer,” 66.7% (n=962) for “contact the shop from where you purchased it,” 12% (n=173) for “upload it on SNS [social networking site],” and 69.8% (n=1007) for “dispose of it.” The majority of respondents answered “contact the manufacturer,” while “eat it without concern” was less than 10%. Interestingly, about 12% of the respondents indicated that they would “upload it on SNS.”

The results of the questions “The frozen food you purchased has a bad smell (rotten, chemical, etc.). You...” were as follows: 4.8% (n=69) for “eat it without worrying,” 78.4% (n=1131) for “contact the manufacturer,” 73.6% (n=1061) for “contact the shop from where you purchased it,” 12.6% (n=182) for “upload it on SNS,” and 74.8% (n=1079) for “dispose of it.” The trend of results was similar to that of contamination with foreign substances (eg, metal, hair).

The results of the questions “You find extra item(s), which you had not selected, in the food parcel delivered to you. You...” were as follows: 30.7% (n=443) for “eat it without worrying,” 75.6% (n=1090) for “contact the shop from where you had ordered it,” 7.3% (n=105) for “upload it on SNS,” 13.1% (n=189) for “dispose of only the extra item(s),” and 7.6% (n=110) for “dispose of all the items.” Interestingly, compared to the previous two questions, the percentage of respondents answering “eating it without worrying” increased. On the other hand, the percentage of respondents indicating that they would “upload it on SNS” decreased.

The results of the logistic regression analysis are presented in Tables 1-3. Male participants and the younger generation were more likely to respond that they would “eat it without worrying” in case of “contamination with foreign substances” (Table 1), “bad smell” (Table 2), or “extra item(s)” (Table 3) in the purchased product. A similar trend was observed for “upload it on SNS,” although the tendency for “extra item(s)” was characteristically higher among “I am sociable” and “brand-conscious” respondents.

**Table 1:** Results of stepwise regression analyses for frozen food purchases containing foreign substances (metal and hair).

		Odds ratio (95% CI)	*P* value
**Sex**			
	Male	2.48 (1.60-3.83)	<.001
	Female	Reference	
**Manufactured domestically**			
	Yes	0.49 (0.31-0.77)	.002
	No	Reference	

**Table 2:** Results of stepwise regression analyses for frozen food purchases with a bad smell.

		Odds ratio (95% CI)	*P* value
**Sex**			
	Male	1.89 (1.05-3.39)	.03
	Female	Reference	
**Age group (years)**			
	15-19	2.81 (1.07-7.36)	.04
	20-39	2.54 (1.15-5.61)	.02
	40-59	1.37 (0.57-3.26)	.48
	60-79	Reference	
**Safety**			
	Yes	0.26 (0.13-0.52)	<.001
	No	Reference	

**Table 3:** Results of stepwise regression analyses for receiving food items not selected by the subjects in the delivered food parcel.

		Odds ratio (95% CI)	*P* value
**Sex**			
	Male	1.48 (1.14-1.92)	.003
	Female	Reference	
**Age group (years)**			
	15-19	7.09 (4.48-11.24)	<.001
	20-39	3.20 (2.24-4.55)	<.001
	40-59	1.87 (1.30-2.70)	.001
	60-79	Reference	
**Manufactured domestically**			
	Yes	0.55 (0.39-0.76)	<.001
	No	Reference	

## Discussion

### Principal Results

This study revealed that awareness of *food defense* as a term is very low compared to that of *food hygiene*. In addition, the percentage of respondents who answered that they would eat their purchased food without worry despite knowing there was something wrong with it was higher among the younger generation, especially male respondents. Similarly, the percentage of respondents who said they would post an article on SNS if something was wrong with the food they had purchased was also higher among the younger generation, especially respondents. Although businesses that manufacture or sell food have recently begun taking food defense measures (ie, protecting food from intentional contamination), the results of this study indicate that if food that has been intentionally contaminated with a foreign substance is sold and delivered to consumers, those consumers could eat it and suffer from health problems. In particular, the results showed that 30.7% (n=443) of the respondents answered that they would “eat without worrying” even if there were products in the package other than those they had purchased. This finding indicates it might be better to assume not only food hygiene issues with the manufacturer but also food defense issues in widely available food delivery services. Our study also demonstrates the importance of food protection measures in food delivery businesses, as well as the consumers’ role in ensuring safety. Promoting consumer education and awareness regarding food defense can contribute to enhancing food safety throughout the food chain.

To protect consumers from health hazards caused by food, food safety has been a matter of concern [8]. Food safety mainly deals with issues such as food poisoning, food additives, and pesticide residues, which can be prevented by avoiding system errors. In other words, food safety can be maintained by creating and following rules for the prevention of food poisoning and additives. In Japan, introducing the hazard analysis and critical control points system has helped maintain and improve food sanitation management throughout the food supply chain. Hazard analysis and critical control points–based hygiene management has become mandatory for all food businesses as of 2021 [16]. In recent years, food hygiene measures have been implemented not only by food manufacturers and sellers but also by consumers at home. For example, the World Health Organization [17] advocates the following core messages as the “Five Keys to Safer Food”: (1) keep clean, (2) separate raw and cooked, (3) cook thoroughly, (4) keep food at safe temperatures, and (5) use safe water and raw materials.

In light of recent incidents of intentional contamination of food with foreign substances, food manufacturing companies must implement food defense measures [10,13-15,18]. In addition to food manufacturing companies, businesses that serve food directly to consumers, such as restaurants and stores, are likely to be targets of intentional contamination by foreign substances. Intentional contamination in restaurants and other food service businesses is characterized by the fact that food is prepared in the kitchen and delivered to consumers immediately. If a foreign substance is intentionally mixed into the food provided by a restaurant, although the possibility of a widespread health hazard is not high, the possibility of health damage is high because the foreign substance is mixed into the food just before it reaches the consumer. Therefore, restaurants and stores must implement food safety measures. Home delivery of food products has recently become a common practice. Businesses that provide home delivery of food products must also consider measures to ensure the safety of food products during home delivery as an aspect of food defense.

Our study showed that young people, especially young men and boys, are more likely to eat their purchased food even when something is wrong with it. In addition, younger generations, especially young men and boys, are more likely to eat even if they receive extra food they did not order. Unfortunately, health hazards are more certain when foreign substances are intentionally mixed during food delivery because it occurs just before the food reaches a consumer. In addition, there is a possibility of individuals causing health hazards for consumers by replacing legitimate finished products with those contaminated with foreign substances or adding a finished product with a foreign substance to a legitimate finished product during food delivery. Therefore, food delivery companies must adopt food defense measures, and strengthen them through the establishment of rules. For example, it is necessary to ensure that delivery personnel do not leave food unattended, even temporarily, during the delivery process. In Japan, home delivery services for prescription drugs were also implemented during the COVID-19 pandemic. Although this study was conducted on food products, the participants’ response of “ingesting without concern even if there is an extra item” indicates a risk for delivery services in other industries. In addition, consumers also need to protect themselves and their families from food-related health hazards by thoroughly checking whether the purchased food has any abnormalities or if its condition is different than usual. Consumers, as well as food manufacturers and sellers, need to be aware that they play a role in preventing health hazards caused by intentional food contamination.

### Limitations

This study has several limitations that must be acknowledged. First, it should be noted that this survey was conducted on the web. This may have influenced respondents’ tendency to answer “upload it to SNS” in case of foreign material contamination. The data were collected only from respondents registered with an web-based panel company, thus, there may have been bias. Nonetheless, panel-based surveys have been widely used in recent years [19-23]. Second, because the participants in this study were recruited using an equal allocation of men and women by age group, they may not be representative of the country’s population by age group or place of residence as a whole. Third, the timing of the survey may have been influenced by the fact that it was conducted during the COVID-19 pandemic state of emergency. Fourth, the participants received a small cash reward for responding to the survey, which may have affected the randomness of the sample. However, since the research firm built the panel for the survey based on large enrollment, bias is expected to be minimized.

### Conclusions

The findings of this study indicate that if food intentionally contaminated with a foreign substance is sold and delivered to consumers, it is possible that consumers will eat it and experience health problems. Therefore, it is crucial for not only food manufacturers but also food delivery service providers to consider food defense measures. Additionally, promoting consumer education and awareness regarding food defense can contribute to enhancing food safety throughout the food chain.

## Abbreviations

SNS: social networking site

## Data Availability

The data used in this study are available from the corresponding author upon request.
